# Bayesian estimation of diagnostic accuracy of fecal culture and PCR-based tests for the detection of *Salmonella enterica* in California cull dairy cattle

**DOI:** 10.7717/peerj.8310

**Published:** 2020-01-15

**Authors:** John M. Adaska, Pius S. Ekong, Kristin A. Clothier, Deniece R. Williams, Paul V. Rossitto, Terry W. Lehenbauer, Edward R. Atwill, Xunde Li, Sharif S. Aly

**Affiliations:** 1 School of Veterinary Medicine, University of California, California Animal Health and Food Safety Laboratory System, Tulare, CA, United States of America; 2 School of Veterinary Medicine, University of California, Veterinary Medicine Teaching and Research Center, Tulare, CA, United States of America; 3 Department of Population Health and Reproduction, School of Veterinary Medicine, University of California, Davis, CA, United States of America; 4 School of Veterinary Medicine, University of California, Western Institute for Food Safety and Security, Davis, CA, United States of America

**Keywords:** Bayesian, Diagnostic accuracy, Sensitivity, Specificity, *Salmonella*, Dairy cow, Culture, PCR, California, Feces

## Abstract

Epidemiological studies of low prevalence disease problems are often hindered by the high cost of diagnostic testing. The objective of this study was to evaluate PCR screening of both individual and pooled fecal samples from culled dairy cows for the *inv*A gene of *Salmonella* followed by culture to determine if the sensitivity and specificity were comparable to the results from traditional culture methods applied to individual samples. Cows from six different dairies were sampled in all four seasons. A total of 240 individual cow fecal samples, 24 fecal pools and 24 pools of 24-hour tetrathionate enrichment broth were tested. Diagnostic sensitivity of PCR screening followed by culture of PCR positive or indeterminate samples (i.e PCR-CUL method) was lower than that of culture (CUL) when applied to individual fecal samples (94.8%, 99.5%), however the specificity was comparable (99.6% and 97.7% respectively). For pools of five fecal samples and pools of five, 24 h tetrathionate broth samples, the specificity of both tests were comparable (∼98%); however, their sensitivity was only comparable in pooled fecal samples (∼93%) but greater for culture compared to PCR-CUL in pooled broth samples (∼99% versus ∼93%). Compared to culture results from testing of individual fecal samples, testing pooled fecal samples by culture had a relative sensitivity of 74% and relative specificity of 96%, testing pooled fecal samples by PCR-CUL resulted in relative sensitivity of 90% and relative specificity of 96%. Testing of pooled 24-hour enrichment broth by PCR-CUL increased the relative sensitivity and specificity to 100%. PCR testing followed by culture of positive or indeterminate samples is a time saving alternative to traditional methods. In addition, pooling of samples may be a useful method for decreasing cost if study aims can accommodate a moderate loss of relative sensitivity.

## Introduction

Nontyphoidal *Salmonella enterica* infections are estimated to be the leading cause of foodborne illnesses in the United States resulting in over a million cases, about 20,000 hospitalizations, and more than 400 deaths annually (Scallan et al., 2011). Foods of animal origin are important sources of *Salmonella* infections in humans (Buncic & Sofos, 2012; Pires et al., 2009; Scallan et al., 2011). In a 2002 multi-state study in the US, Varma et al. (2006) identified consumption of undercooked ground beef as a risk factor for infection with multi-drug resistant *Salmonella* Newport infection. About 18% of ground beef produced in the United States are sourced from cull dairy cows (NAHMS, 1996). Beef carcass contamination with *Salmonella* sp. may occur during slaughter. The prevalence of *Salmonella* sp. in cull dairy cattle in the US had been reported to range from 0.0% to 93.0% depending on the season and day of the week that the samples were collected (Troutt et al., 2001).

Traditionally, the prevalence of *Salmonella* sp. has been determined using individual sample culture methods. However, these methods take several days to complete and require the culturing of a large number of samples if the prevalence of *Salmonella* is low. Singer et al. (2006) showed that the use of PCR on pools of five fecal samples can improve the speed and efficiency of detecting *Salmonella* spp. in dairy cattle feces. However, the study was not conducted on cull dairy cattle and had several limitations, including non-random selection of both cattle in the study and the individual samples included in each pool; not culturing pooled samples; and not serotyping *Salmonella* sp. isolates (Singer et al., 2006). Abu Aboud et al. (2016) in a study conducted on seven California dairies, estimated the crude and seasonal prevalence of *Salmonella* fecal shedding in cull dairy cattle based on pooled and individual fecal culture. In this current study, we estimate the prevalence of *Salmonella* fecal shedding in California cull dairy cattle based on the traditional culture (CUL) and a combined PCR-culture (PCR-CUL) tests using individual cow fecal samples (IF), pools of five fecal samples (FP), and pooled enrichment broths from five fecal samples after 24 h of incubation (BP). In addition, we estimated and compared the diagnostic accuracy of CUL and PCR-CUL tests for the detection of *Salmonella enterica* in IF, FP, and BP samples.

## Materials and Methods

### Fecal sampling

Individual fecal samples were collected from each of 240 cull dairy cattle from 6 dairies in the San Joaquin Valley of California between 2015 and 2016, specifically during summer (August–September), fall (October–November), winter (January–March), and Spring (April–May). The characteristics of the dairies are provided in Table 1 and the survey-weighted proportion for culling reasons are included in Table 2. Each dairy was sampled quarterly over a 12 month period to control for seasonal effects. At each sampling event, individual fecal samples were collected rectally, using clean palpation sleeves, from 10 cull cows within 24 h prior to sale from the dairy. For herds culling more than 10 cows on a given sampling date, a predetermined random number list was used to select the 10 cows for sampling. Samples were transferred to 2 oz plastic snap cap milk tubes immediately after collection and transported on ice to the Diary Epidemiology Laboratory, Veterinary Medicine Teaching and Research Center, Tulare, CA.

**Table 1:** Characteristics of six California dairy herds enrolled in a cross-sectional study to survey for *Salmonella* sp. fecal shedding in a random sample of cull dairy cows.

**Dairy**	**Mean milking herd size (SE)**	**RHA**,^a^**Kg (SE)**	**Herd breed**^b^**distribution, (%)**	**Herd percent culled per month, % (SE)**	**Culling times per month (SE)**	**Percent of cull sold as beef, %**	**Facility design**^c^
1	3,725 (25)	12,079 (297)	H (38.5%), J (61.5%)	4.93 (0.76)	2.13 (0.13)	100.00	FS
2	2,938 (63)	12,084 (138)	H (100%)	4.95 (0.23)	3.5 (0.29)	100.00	FS
3	2,807 (110)	9,107 (200)	J (100%)	4.6 (1.19)	2.13 (0.13)	84.00	DL
4	5,350 (87)	14,440 (647)	H (97%), J (3%)	8.23 (0.95)	2.5 (0.20)	100.00	FS
5	2,550 (87)	10,784 (140)	H (100%)	3.06 (0.09)	1.0 (0.0)	100.00	DL
6	1,525 (14)	14,895 (292)	H (100%)	4.28 (1.47)	1.67 (0.12)	100.00	DL
All	3,149 (24)	12,231 (207)	H (72.6%), J (27.4%)	5.01 (0.37)	2.15 (0.04)	97.33	

**Notes.**

a Rolling herd average defined as the mean milk produced per milking cow in the herd in 365 days.

b (H), Holstein and (J), Jersey breeds.

c Facility design: (FS), freestall and (DL), drylot.

**Table 2:** Survey-weighted proportion for culling reasons for 239 cows on six California dairies surveyed over a course of a year.

**Culling reason**	**Season**^a^										***P* value**
	**Summer (*N* = 60)**		**Fall (*N* = 60)**		**Winter (*N* = 59)**^b^		**Spring (*N* = 60)**		**Overall (*N* = 239)**^c^		
	**N**	**% (SE)**	**N**	**% (SE)**	**N**	**% (SE)**	**N**	**% (SE)**	**N**	**% (SE)**	
Low milk production	40	59.88% (5.73)	37	58.79% (5.18)	35	69.09% (4.79)	44	78.47% (3.52)	156	65.87% (2.50)	0.081
Poor reproduction	28	52.43% (6.25)	20	40.52% (5.97)	8	16.46% (4.61)	18	31.97% (5.26)	74	35.03% (2.79)	0.010
Lameness	7	10.18% (2.97)	8	15.29% (4.71)	3	4.05% (1.87)	7	10.65% (3.32)	25	10.01% (1.73)	0.151
Mastitis	0	0.00% (-)	7	14.14% (4.67)	10	11.40% (3.10)	7	8.98% (2.19)	24	8.93% (1.63)	0.055
Other^d^	14	21.65% (4.77)	18	27.91% (5.32)	20	32.66% (5.55)	11	15.62% (3.57)	63	25.23% (2.53)	0.166

**Notes.**

a Study year and seasons included summer (July 1–September 30, 2015), fall (October 1–December 31, 2015), winter (January 1–March 31, 2016) and spring (April 1–June 30, 2016).

b Due to sample ID labelling error, 1 sample was not reconciled with dairy record (Herd 5, winter sample).

c Totals and percents do not add up to 239 or 100%, respectively, due to multiple cull reasons.

d The category labeled other reasons included the following conditions: unknown illness, gastrointestinal disorder, poor udder confirmation, undiagnosed fever, pneumonia or eye disease.

### Pooling and enrichment

For each IF sample, approximately 8 g of feces were transferred to a sterile 15 ml polypropylene tube for individual culture. A predetermined random number list was used to select individual samples that were incorporated into each 5 sample pool and 2 g of feces from each of the 5 IF samples were transferred to a 50 ml sterile polypropylene tube (total of 10 g of feces each) and thoroughly mixed to create a FP of five samples. IF and FP samples were submitted to the CAHFS Tulare laboratory on the day of collection. Laboratory personnel were blinded to the dairy of origin and the individual samples contained in each pool. One gram of feces from either IF or FP samples were placed into a tetrathionate (TT) selective enrichment broth containing 0.01% brilliant green and 0.02% iodine at a 1:10 sample-to-broth ratio, incubated for 18–24 h at 37 ± 2 °C. Following incubation 0.75 ml of broth from each of the IF samples were combined to create broth pools (BP) from 5 individual fecal samples. The selection of individual broth samples for pooling followed the random selection performed for the fecal pools. A total of 14 cultures and 14 PCR tests were performed for each group of 10 fecal samples collected from a given dairy on a given day: Ten IF samples, two FP samples and two BP samples. The FP and the BP were comprised of the same IF samples.

### Traditional culture (CUL)

An aliquot of incubated TT broth from each IF, BP and FP sample was inoculated onto two culture media (minimally-selective xylose lysine deoxycholate [XLD] plate and a highly-selective Xylose Lactose Tergitol *4* [XLT-4] plate) and incubated for 18–24 h at 37 ± 2 °C. Up to three H_2_S positive, *Salmonella* suspect colonies from each set of plates were subcultured separately, onto a 5% sheep blood agar-MacConkey agar bi-plate which was incubated for 18–24 h at 37 ± 2 °C. One colony from each biplate was used for biochemical testing which included triple sugar iron (TSI), urea, motility indole ornithine (MIO), citrate, O-nitrophenyl-beta-D-galactopyranoside (ONPG), and lysine iron agar (LIA) slants. Individual colonies with compatible biochemical test results (Quinn, 2011) were identified to the serogroup (Difco antiserum, BD Diagnostics, Sparks, MD) and serovar (*Salmonella* antisera, Statens Serum Institut, Denmark) level as described using the White–Kauffmann–Le Minor scheme (Grimont & Weill, 2007).

### Salmonella PCR (PCR-CUL)

PCR testing of IF, FP and BP for the detection of *Salmonella* spp. was performed in a 96 well format by the CAHFS Davis bacteriology laboratory. Testing was based on the *invA* gene target as described previously (Suo et al., 2010) with the inclusion of an exogenous internal positive control (IPC) DNA (TaqMan Exogenous Internal Positive Control, Life Technologies, Carlsbad, CA). PCR results were defined as positive when both the *invA* and IPC crossed the threshold, negative when *invA* failed to cross the threshold but IPC crossed at ≤ 35 Ct, and inconclusive (failed internal positive control) when *invA* failed to cross the threshold and IPC either failed to cross threshold or crossed at >35 Ct. Negative samples were not tested further. Broth samples that were positive or inconclusive by PCR were subcultured onto one minimally selective (MacConkey agar [MA]) and two highly-selective (Hektoen enteric agar [H]) and brilliant green with 0.002% novobiocin agar [BGN] culture media. After incubation for 18–24 h at 37 ± 2 °C, plates were examined for *Salmonella*-suspect colonies, which were confirmed by biochemical testing, as described above. Matrix-assisted laser desorption/ionization time-of-flight (MALDI-TOF) mass spectrometry was performed on colonies from about half of the PCR inconclusive or positive IF (44/87), FP (12/25) and BP (11/25) samples. Individual *Salmonella* colonies were serogrouped and serotyped as described above. Final positive PCR results were defined by the serovar detected.

## Statistical Analysis

### Estimation of diagnostic sensitivity and specificity of tests

The diagnostic sensitivity and specificity of CUL and PCR-CUL for the detection of *Salmonella* sp. based on IF, FP, and BP were estimated using Bayesian no gold standard models as described by Hui & Walter (1980). We assumed conditional dependence between CUL and PCR-CUL because the PCR-CUL method included a culture confirmation following initial screening by PCR. Conditional dependence was modeled and estimated as conditional correlation according to (Dendukuri & Joseph, 2001; Gardner et al., 2000; Georgiadis et al., 2003). To adjust for the study’s survey sampling the data was expanded based on the sampling weights (total number of cows available for sale on a given sampling day/number of cows randomly identified and sampled). External prior information for the model parameters were specified using beta distributions. A non-informative prior (dbeta(1,1)) was specified for all model parameters except for the specificity of CUL which was considered high (mode = 99%; 5th percentile = 97%) (Ekong et al., 2017). In addition, for the individual cow model, the prior for PCR-CUL sensitivity was set with a mode of 85.0% and 5th percentile of 50%, and PCR-CUL specificity was set with a mode of 95% and 5th percentile of 50% based on expert opinion. All models were fitted using three chain models, each consisting of 100,000 iterations with burn-in period of 10,000 iterations. Model convergence was assessed by visual inspection of the Gelman–Rubin diagnostic plots, deviance information criterion (DIC) and effective number of parameters estimated (*p*D).

### Estimation of relative sensitivity and specificity of pooling

The relative sensitivity of pooling of fecal or broth samples was estimated as:

\begin{eqnarray*} \frac{\mathrm{Number~ of~ test~ positive~ pools~ containing~ atleast~ one~ positive~ individual~ fecal~ sample}}{\mathrm{Predicted~ number~ of~ test~ positive~ pools~ based~ on~ positive~ individual~ fecal~ sample}} \end{eqnarray*}

where test positive is CUL or PCR-CUL positive for a known serovar of *Salmonella*. The relative specificity of pooling of fecal or broth samples was similarly estimated as:

\begin{eqnarray*} \frac{\mathrm{Number~ of~ test~ negative~ pools~ containing~ all~ negative~ individual~ fecal~ samples}}{\mathrm{Predicted~ number~ of~ test~ negative~ pools~ based~ on~ negative~ individual~ fecal~ sample}} . \end{eqnarray*}

The Bayesian analyses were conducted using OpenBUGS® version 3.2.3 rev 1012, calculation of relative sensitivity and specificity were conducted using Stata 14.2 (College Station, TX).

## Results

Of the 240 individual fecal samples 60 were positive for *Salmonella* sp. on culture (CUL). Of the 60-culture positive individual fecal samples, 54 were positive for *Salmonella* sp. by PCR –and follow-up culture (PCR-CUL), 4 were PCR positive, follow-up culture negative, and 2 were negative on PCR. Eighteen (18) FP were positive by both culture and by PCR-culture while 30 pools were negative on both. Nineteen (19) BP were positive on both culture and PCR-culture, 28 were negative on both and 1 was positive by culture but negative by PCR-culture (Table 3). The survey weighted results are presented in brackets in Table 3. For the pooled samples, 24/48 pools had no positive samples on IF culture or PCR-CUL and FP and BP were negative on both culture and PCR-CUL. Fifteen of 48 (15/48) pools had 1 or more IF positive on culture with the same number of positive on IF PCR-CUL and these pools had both FP and BP positive on both culture and PCR-CUL. Two of 48 pools (2/48) had 1 or 3 IF samples positive on both culture and PCR-CUL while the FP sample was negative on both culture and PCR-CUL, but the BP was positive on both culture and PCR-CUL (pools 11 and 48). Three of 48 (3/48) pools had 1 IF sample that was positive on culture but negative on PCR-CUL with all FP and BP testing being negative (pools 14, 21 and 39). Two of 48 (2/48) pools had 5 IF samples positive on culture with only 4 IF positive on PCR-CUL but all FP and BP testing was positive (pools 24, 38). One of 48 (1/48) pools had no samples positive on IF testing, FP was positive by both culture and PCR, but BP was negative on both. Lastly, one of 48 (1/48) pools had 1 IF sample that was positive on culture but negative on PCR, all FP testing was negative, but BP was positive by culture and negative by PCR. MALDI testing on 18 IF that were PCR positive or inconclusive but which did not have *Salmonella* sp. isolated on culture identified a variety of bacteria including *Citrobacter amalonaticus* (6 samples), *Citrobacter koseri* (1), *Citrobacter freundii* (2) *Citrobacter* spp. (5), *Proteus mirabilis* (4), *E. coli* (2), *Enterobacter aerogenes*, *Enterobacter* spp., *Klebsiella pneumoniae*, *Klebsiella* spp., and *Leclercia adecarboxylata* (1 each). MALDI testing on PCR positive or inconclusive but *Salmonella* culture negative BP (1 pool) and FP (4 pools) identified similar organisms. *Salmonella* sp. isolates were serogrouped and there were 4, 49, 2, 1 and 5 serogroup B, C1, C2, D1 and grouping sera negative isolates, respectively. The serogroup D1 isolate was presumed to be *Salmonella* Dublin and was not serotyped. Serotyping of isolates in other serogroups identified *S. Typhimurium* (4 isolates), *S. Mbandaka* (1), *S. Montevideo* (47), *S. Rissen* (1), *S. Bardo* (2), *S. Cerro* (4) and 1 untypeable isolate.

**Table 3:** Tabulated detection results^a^ and survey weighted detection results^b^ (in bracket) for *Salmonella* sp. shed in feces of culled cows sampled on six California dairies based on culture and combined PCR-culture testing of enriched brot.

**Sample type**	**Test**^c^		**Number of samples**						**All Herd**
	**Culture**	**PCR-Culture**^d^	**Herd 1**	**Herd 2**	**Herd 3**	**Herd 4**	**Herd 5**	**Herd 6**	
Individual cow	+	+	0 (0)	0 (0)	3 (11)	21 (104)	2 (3)	28 (65)	54 (183)
	+	−	1 (4)	1 (2)	1 (4)	1 (6)	1 (2)	1 (3)	6 (21)
	−	+	0 (0)	0 (0)	0 (0)	0 (0)	0 (0)	0 (0)	0 (0)
	−	−	39 (136)	39 (68)	36 (95)	18 (80)	37 (55)	11 (32)	180 (466)
Fecal pool	+	+	0 (0)	0 (0)	3 (10)	6 (29)	2 (3)	7 (17)	18 (59)
	+	−	0 (0)	0 (0)	0 (0)	0 (0)	0 (0)	0 (0)	0 (0)
	−	+	0 (0)	0 (0)	0 (0)	0 (0)	0 (0)	0 (0)	0 (0)
	−	−	8 (28)	8 (14)	5 (12)	2 (9)	6 (9)	1 (3)	30 (75)
Broth pool	+	+	0 (0)	0 (0)	2 (7)	8 (38)	2 (3)	7 (17)	19 (65)
	+	−	0 (0)	0 (0)	1 (4)	0 (0)	0 (0)	0 (0)	1 (4)
	−	+	0 (0)	0 (0)	0 (0)	0 (0)	0 (0)	0 (0)	0 (0)
	−	−	8 (28)	8 (14)	5 (11)	0 (0)	6 (9)	1 (3)	28 (65)

**Notes.**

a Actual data representing the total number of cows randomly identified and sampled at each dairy.

b Data was expanded based on the sampling weights (total number of cows available for sale on a given sampling day/number of cows randomly identified and sampled) to adjust for the studys survey sampling.

c +, Positive; −, Negative.

d Results of enriched broth of fecal samples tested in series using PCR for *Salmonella invA* gene followed by culture when testing positive or inconclusive by PCR.

The Bayesian survey-weighted estimates of *Salmonella* sp. true prevalence in cull dairy cattle feces varied among the study herds ranging from 0.53% (95% PI: 0.01–2.78) to 67.3% (95% PI: 57.6–76.1) during the study period (Table 4). Furthermore, the estimated true prevalence varied by season ranging from 20.7% to 35.2% (Table 4). At the level of IF samples, diagnostic sensitivity of culture exceeded that of PCR-CUL (99.5% versus 94.8%) however the specificity was comparable (97.7% and 99.6% respectively). For FP and BP samples the specificity of both tests were comparable (∼98%), however, their sensitivity was only comparable in FP samples (∼93%) but greater for culture compared to PCR-CUL in BP samples (∼99% versus ∼93%) (Table 5).

**Table 4:** Bayesian-adjusted survey-weighted true prevalence, by herd and season, of *Salmonella sp*. shed in feces of 240 culled cows sampled on six California dairies (PCR positive or inconclusive and culture positive of enriched broth of fecal samples).

**Dairy**	**Prevalence, % (95% CI)**					**Serogroup**	**Serotype**
	**Summer**^a^	**Fall**	**Winter**	**Spring**	**All Seasons**		
1	0.0 (-)	0.0 (-)	0.0 (-)	0.0 (-)	0.53 (0.01, 2.78)		*S*. sp. untypeable
2	0.0 (-)	0.0 (-)	0.0 (-)	0.0 (-)	1.05 (0.03, 5.47)	C1	*S. Mbandaka*
3	0.0 (-)	8.97 (7.77, 9.91)	19.4 (18.1, 20.6)	0.0 (-)	11.1 (5.97, 18.2)	B	*S. Typhimurium*
						C1	*S. Montevideo*
4	61.2 (59.2, 63.6)	71.7 (69.5, 74.3)	50.8 (48.9, 52.8)	29.9 (28.4, 31.4)	57.0 (49.8, 64.1)	C1	*S. Montevideo*
						C2	*S. Bardo*
5	0.0 (-)	8.97 (7.77, 9.91)	8.97 (7.77, 9.91)	0.0 (-)	6.27 (1.71, 14.5)	B	*S. Typhimurium*
						C1	*S. Rissen*
						D	
6	8.97 (7.77, 9.91)	0.0 (-)	82.2 (79.7, 85.0)	92.6 (89.9, 95.8)	67.3 (57.6, 76.1)	C1	*S. Montevideo*
							*S. Cerro*
All	20.7 (19.4, 21.9)	35.2 (33.6, 36.8)	29.7 (28.2, 31.2)	23.9 (22.5, 25.2)	29.4 (28.4, 30.5)		

**Notes.**

a Study year and seasons included summer (July 1–September 30, 2015), fall (October 1–December 31, 2015), winter (January 1–March 31, 2016) and spring (April 1–June 30, 2016).

**Table 5:** Estimated diagnostic sensitivity and specificity (posterior median and 95% CI) of culture (Culture) and PCR-CUL tests of enriched broth of fecal samples. Diagnostic estimates are based on dependence^a^ Bayesian latent class models for the detection of *Salmonella* sp. shed in feces of culled cows sampled on six California dairies.

**Sample type**	**Tests**	**Sensitivity, %(95% CI)**	**Specificity, %(95% CI)**
Individual cow^b^	Culture	99.5 (97.0, 99.9)	97.7 (96.1, 98.7)
	PCR-CUL	94.8 (90.5, 97.9)	99.6 (98.7, 99.9)
Fecal pool	Culture	93.7 (75.0, 99.8)	98.9 (97.2, 99.7)
	PCR-CUL	93.3 (75.0, 99.4)	98.3 (94.2, 99.7)
Broth pool	Culture	98.6 (92.5, 99.9)	98.7 (96.6, 99.7)
	PCR-CUL	93.2 (84.9, 98.3)	98.3 (93.8, 99.7)

**Notes.**

a Dependence model specifying uniform priors for all parameters except for the specificity of culture.

b Additional priors specified for sensitivity and specificity of PCR-Culture.

Pooling of fecal samples versus broth did not result in differences in sensitivity or specificity for either CUL or PCR-CUL tests with the exception of CUL’s sensitivity for BP being higher than that of FP (98.6% versus 93.7%) (Table 5). Relative diagnostic accuracy for pooling of either fecal or broth samples and for CUL and PCR-CUL are summarized in Table 6. Relative sensitivity and relative specificity estimates for CUL compared to PCR-CUL were comparable with the exception of relative sensitivity for broth pools (87% vs. 83%). Relative specificity of fecal pools compared to broth pools were similar for both CUL and PCR-CUL. However, relative sensitivity for CUL of broth pools was greater than that of fecal pools.

**Table 6:** Estimated relative sensitivity^a^ and specificity^b^ (mean and 95% CI) of enriched broth culture (CUL) versus PCR-CUL for the detection of *Salmonella* sp. in fecal pools (FP), or broth pools (BP) of five samples from culled cows sampled, compared to results from testing of individual samples.

**Individual sample diagnostic test**	**Relative Sensitivity, % (95% CI)**		**Relative Specificity, % (95% CI)**	
	**FP CUL**	**FP PCR-CUL**	**FP CUL**	**FP PCR-CUL**
CUL^c^	73.9 (51.6, 89.8)	73.9 (51.6, 89.8)	96.0 (79.6, 99.9)	96.0 (79.6, 99.9)
PCR-CUL^d^	89.5 (66.9, 98.7)	89.5 (66.9, 98.7)	96.6 (82.2, 99.9)	96.6 (82.2, 99.9)
	**BP CUL**	**BP PCR-CUL**	**BP CUL**	**BP PCR-CUL**
CUL^c^	87.0 (66.4, 97.2)	82.6 (61.2, 95.0)	100.0 (86.3, 100)	100.0 (86.3, 100)
PCR-CUL^d^	100.0 (82.4, 100)	100.0 (82.4, 100)	96.6 (82.2, 99.9)	100.0 (88.1, 100)

**Notes.**

a Relative sensitivity defined as proportion of pools correctly identified as positive (pool contained at least 1 positive sample).

b Relative specificity defined as proportion of pools correctly identified as negative (pool contained all negative samples).

c Comparison based on individual fecal sample CUL.

d Comparison based on individual fecal sample PCR-CUL.

## Discussion

PCR testing is a relatively modern technique that offers a significant improvement in turnaround times when applied to routine laboratory diagnostic testing. Similarly, the use of sample pooling when testing large populations with low disease or pathogen prevalence serves to improve study economics by decreasing the overall amount of testing that needs to be done to identify affected individuals (Aly et al., 2012; Murai et al., 2014). In the study reported here, we evaluated a diagnostic approach that utilized both PCR testing and sample pooling as applied to an epidemiological study of *Salmonella* in cull dairy cattle in California. A total of 18 IF samples were positive or inconclusive for *Salmonella* by PCR but an isolate could not be recovered. The inability to isolate *Salmonella* from these samples may have been due to low numbers of *Salmonella*, inactivated strains, or non-viable *Salmonella*; or recovery may have been compromised by other bacteria in the sample (e.g., *Citrobacter* sp, swarming *Proteus*). It is also possible that the bacteria isolated in these samples cross-reacted on the PCR to yield false positive results. The addition of the culture step following the screening PCR may have helped increase the specificity of the PCR-CUL testing.

Posterior estimates of diagnostic sensitivity and specificity for CUL and PCR-CUL methods for the detection of fecal *Salmonella* in adult dairy cows showed high and comparable results for IF samples, FP, and BP samples. The diagnostic specificity of CUL and PCR-CUL tests observed in the current study are comparable in magnitude to those recorded for culture and PCR methods for the detection of other foodborne fecal pathogens, *Escherichia coli* O157 and non-O157 serogroups (Ekong et al., 2017; Ekong et al., 2018). In the Ekong et al. (2017) and Ekong et al. (2018) studies, diagnostic sensitivity of the quantitative PCR method was greater than that of culture. In this current study, diagnostic sensitivity of CUL was slightly greater than that of PCR-CUL, however, their 95% confident intervals overlapped. Conversely, the diagnostic specificity of CUL and PCR-CUL in this study were comparable to those seen in the Ekong et al. (2017) and Ekong et al. (2018) studies. While pooling decreased sensitivity for both tests, testing FP and BP by PCR-CUL produce comparable estimates of sensitivity, pending further testing of the IF sample constituting the positive pools. Subsequently, identifying individual samples that are positive for *Salmonella* will be subject to the sensitivity and specificity of the individual sample test chosen. A second option which may offer cost saving is pooling fecal samples compared to broth samples. However, investigators should expect a decrease in sensitivity due to pooling of fecal compared to broth samples as estimated from current study. A higher sensitivity of BP may be explained by propagation of *Salmonella* in enriched broth. This decrease in sensitivity is of lesser magnitude when testing using PCR-CUL compared to CUL alone (90% vs. 74%). The estimated mean relative sensitivity of CUL for FP and BP in this study are slightly higher and had less variability as compared to the estimates reported in the study of Abu Aboud et al. (2016) which may be explained by differences over time despite being the same herds. However, these studies produced comparable estimates (mean and 95% CI) for relative specificity of CUL method.

Overall, there was high variability in the estimated prevalence of *Salmonella* in feces of cull dairy cows across the study herds. Lower prevalence (ranging from 0.53% to 11.1%) was recorded on four dairies, while high prevalences (57.0% and 67.3%) were recorded on 2 dairies. The lower prevalence observed on some dairies in this study is similar to estimates recorded in previous studies of dairy cattle across U.S. and in California. Wells et al. (2001) reported an overall prevalence of fecal shedding of *Salmonella* of 5.4% in milking cows and 18.1% in cows to be culled across US dairies and 14.9% for cull dairy cows at markets across the US. In a 2007 study, Lombard et al. (2012) reported an overall fecal *Salmonella* prevalence in individual cows of 14% in dairies across the U.S., with a prevalence of 3.9% in the West region of the US. In addition, Blau et al. (2005) in a 2002 study of dairy herds across 21 states of the US, reported a fecal *Salmonella* prevalence of 7.3% in milk cows. However, none of these previous studies were adjusted for test imperfection.

Abu Aboud et al. (2016) reported fecal *Salmonella* prevalence ranging from 1.97% to 7.11% from a study of 249 cull cows sampled on 7 California dairies, 6 of which were the same herds in the current study. In comparison, the same herds in our study had a *Salmonella* prevalence of 21% to 35% across seasons. Differences in *Salmonella* prevalence between both studies may be related to the increase in rainfall during the current study’s period compared to the drought experienced during Abu Aboud’s study period (2016). Specifically, these herds had lower mean seasonal rainfall in the earlier study’s fall (1.0 cm versus 4.7 cm) and spring (0.6 cm versus 0.9 cm); despite greater rainfall in the winter compared to the current study (3.2 cm versus 1.5 cm) (https://www.cnrfc.noaa.gov/rainfall_data.php). Other reasons for the high variability in estimated fecal prevalence of *Salmonella* across the study dairies and seasons may be due to herd changes over time and heat stress during the summer which may explain the increase in prevalence over the summer months in absence of rainfall. Additionally, differences in management practices across dairies may greatly impact disease prevalence.

The study showed that PCR screening followed by culture (PCR-CUL) of any PCR positive or indeterminate samples had similar diagnostic sensitivity and specificity to culture when applied to individual fecal samples, pools of five fecal samples or pools of five 24-hour enrichment (TT) broth samples for the detection of *Salmonella* sp. However, pooling either fecal samples or 24-hour enrichment broth generally yielded lower relative sensitivity but comparable specificity to testing individual samples by culture for the detection of *Salmonella* sp.

## Conclusions

Our study showed that CUL and PCR-CUL produced high and comparable performance for detection of *Salmonella* in feces of cull dairy cows when applied to individual fecal samples, fecal pools, and enriched broth pools. PCR-CUL is a time saving alternative to traditional methods. In addition, pooling of samples may be a useful method for decreasing cost if study aims can accommodate a moderate loss of relative sensitivity. The results indicate that PCR screening of pooled 24-hour enrichment broth samples followed by culture of individual broth samples from the constituents of those pools should be considered as a testing approach for the detection of *Salmonella* sp. in cattle feces when costs are a consideration.

##  Supplemental Information

10.7717/peerj.8310/supp-1 Supplemental Information 1 Analysis code broth pools

10.7717/peerj.8310/supp-2 Supplemental Information 2 Analysis code fecal pools

10.7717/peerj.8310/supp-3 Supplemental Information 3 Analysis code individual fecal samples

## Additional Information and Declarations

### Data Availability

The following information was supplied regarding data availability: The de-identified data was provided for peer review only as the dairy owners did not consent to publishing it alongside the article.
